# Supplementary material for: Understory Vegetation Change Following Woodland Reduction Varies by Plant Community Type and Seeding Status: A Region-Wide Assessment of Ecological Benefits and Risks
Source: Plants (Basel). 2020 Aug 28;9(9):1113. doi: 10.3390/plants9091113 (PMC7570382; doi:10.3390/plants9091113)
Supplement: Supplementary file 1 [file plants-09-01113-s001.pdf]

Table S1. Analysis of variance (ANOVA) results for the effects of P-J reduction treatment and plant community type on pre-treatment vegetation and ground cover variables. Note: variables that could not be transformed to meet ANOVA assumptions were analyzed for main-, but not interaction-effects (i.e., indicated by dashes) with non-parametric Kruskal-Wallis tests. Asterisks indicate significance (****P* < 0.001, ***P* < 0.01, **P* < 0.05).

Effect	Pinyon-Juniper	Sagebrush	Perennial grass	Perennial forb	Annual grass	Annual forb	Bare ground	Cryptogam
Treatment	21.25***	6.69**	6.72**	0.05	0.16	0.052	0.9825	0.60
Community	1.64	2.21	0.70	7.28**	7.66*	0.2422	0.2459	7.44*
T x C	0.56	1.62	1.61	0.30	---	0.5487	1.291	---
